# WHO priority pathogens, ESKAPE bacteria, and antimicrobial resistance surveillance in household wastewater, Gombe, Nigeria

**DOI:** 10.1099/acmi.0.001100.v3

**Published:** 2026-01-19

**Authors:** Zeenatuddeen Muhammad, Muhammed Tukur Adamu, Lawal Garba, Umar Abdullahi Tawfiq, Ibrahim Yusuf

**Affiliations:** 1 Department of Microbiology, Gombe State University, Gombe, Nigeria; 2 Environmental Health Council of Nigeria, Abuja, Nigeria; 3 Department of Microbiology, Bayero University Kano, Kano, Nigeria

**Keywords:** carbapenemase, environmental surveillance, ESKAPE organisms, household wastewater, Nigeria, World Health Organization (WHO) priority pathogens

## Abstract

**Background.** World Health Organization (WHO) priority bacterial pathogens and ESKAPE (*Enterococcus faecium*, *Staphylococcus aureus*, *Klebsiella pneumoniae*, *Acinetobacter baumannii*, *Pseudomonas aeruginosa* and *Enterobacter* spp.) organisms in household wastewater pose critical community transmission risks, yet surveillance data from Sub-Saharan Africa remain limited. This study provides the first comprehensive priority pathogen detection and antimicrobial resistance assessment in household wastewater from Gombe State, Nigeria, focusing on organisms of highest clinical concern.

**Methods.** We conducted targeted surveillance for WHO priority pathogens in 320 household wastewater samples across seven districts in Gombe using multi-stage sampling. Some priority Gram-negative pathogens (*Escherichia coli*, *K. pneumoniae*, *P. aeruginosa* and *Enterobacter* spp.) were specifically isolated and characterized. Antimicrobial susceptibility testing followed Clinical and Laboratory Standards Institute (CLSI) 2024 guidelines across 12 antibiotics targeting critical resistance patterns. Extended-spectrum beta-lactamase (ESBL) and carbapenemase detection focused on priority pathogen isolates, with PCR confirmation of key resistance genes.

**Results.** Priority pathogen detection revealed *E. coli* (131 isolates, 32.6%) as the dominant WHO priority pathogen, followed by *K. pneumoniae* (77 isolates, 19.2%) and *P. aeruginosa* (45 isolates, 11.2%). The ESKAPE pathogen *P. aeruginosa* showed 73.3% multidrug resistance (MDR), with carbapenemase gene detection [Verona integron-encoded metallo-beta-lactamase gene (*bla*VIM), 60%; *K. pneumoniae* carbapenemase gene (*bla*KPC), 20%]. The critical priority pathogen *K. pneumoniae* demonstrated a 79.2% MDR prevalence, with universal beta-lactamase CTX-M gene (*bla*CTX-M) gene presence (100%) among ESBL producers. Priority pathogen *E. coli* exhibited an alarming 84.7% MDR rate, with widespread ESBL production (51.5%). Carbapenem resistance among priority pathogens reached 41.3%, indicating last-resort antibiotic failure in critical organisms.

**Conclusions.** Household wastewater in Gombe harbours WHO priority pathogens and ESKAPE organisms with high antimicrobial resistance prevalence. These preliminary findings suggest substantial environmental circulation of resistant bacteria and highlight the need for enhanced surveillance, further investigation of community transmission risks and strengthened antimicrobial stewardship programmes.

## Data Availability

The datasets generated and/or analysed during the current study will be made available as a supplementary file accompanying this manuscript.

## Introduction

Antimicrobial resistance (AMR) in World Health Organization (WHO) priority pathogens represents one of the most critical global health security threats of the 21st century [1], with the WHO identifying specific organisms requiring urgent research and development of new antibiotics [2,3]. The WHO Global Priority Pathogen List categorizes bacteria into critical, high and medium priority based on their threat level to human health, with critical priority pathogens including carbapenem-resistant *Acinetobacter baumannii*, *Pseudomonas aeruginosa* and Enterobacteriaceae (including *Escherichia coli* and *Klebsiella pneumoniae*) [4].

The ESKAPE (*Enterococcus faecium*, *Staphylococcus aureus*, *K. pneumoniae*, *A. baumannii*, *P. aeruginosa* and *Enterobacter* spp.) pathogens represent organisms that effectively ‘escape’ the effects of antibiotics and are responsible for increasing rates of nosocomial infections [5,6]. Among these, Gram-negative ESKAPE organisms, *K. pneumoniae*, *A. baumannii*, *P. aeruginosa* and *Enterobacter* spp., pose particular concern due to their intrinsic and acquired resistance mechanisms and their ability to rapidly disseminate resistance genes [7].

Environmental surveillance of pathogens has emerged as a critical component of global AMR monitoring [8], with wastewater serving as a concentrated reservoir for community-circulating resistant organisms [9]. Unlike clinical surveillance that captures individual infections, wastewater-based priority pathogen detection provides population-level insights into the community burden of the most dangerous resistant bacteria, potentially identifying emerging threats before their clinical manifestation.

Priority pathogen detection in household wastewater is particularly significant because these environments create ideal conditions for horizontal gene transfer among bacterial populations [10,11]. The convergence of human gut microbiota, pharmaceutical residues and diverse bacterial communities in wastewater systems facilitates the development and dissemination of resistance mechanisms in organisms already identified as critical threats to public health [12].

Sub-Saharan Africa faces a disproportionate burden from priority pathogens [13], yet environmental surveillance data specifically targeting WHO priority pathogens and ESKAPE organisms remain severely limited [14]. Nigeria, with its large population and emerging healthcare infrastructure, represents a critical knowledge gap in understanding community-level circulation of priority pathogens through household wastewater systems.

The detection and characterization of priority pathogens in environmental samples provide essential data for risk assessment, outbreak preparedness and intervention planning [15]. Carbapenemase-producing Enterobacteriaceae, carbapenem-resistant *P. aeruginosa* and multidrug-resistant *A. baumannii* represent organisms of highest concern due to limited therapeutic options and their potential for rapid dissemination [16].

Gombe State presents unique characteristics for priority pathogen surveillance, with diverse population demographics, varying sanitation infrastructure and the potential for priority pathogen circulation through household wastewater systems. Understanding the priority pathogen landscape in Gombe provides critical insights for regional preparedness and contributes essential African data to global priority pathogen surveillance networks.

This study addresses critical knowledge gaps by conducting the first comprehensive priority pathogen detection and surveillance in household wastewater from Gombe State, specifically targeting WHO priority pathogens and ESKAPE organisms. Our findings provide essential baseline data for Nigerian priority pathogen surveillance and demonstrate the feasibility of environmental monitoring for the most dangerous antimicrobial-resistant bacteria in resource-limited settings.

The aim of this study was to conduct comprehensive priority pathogen detection and AMR surveillance in household wastewater from Gombe State, Nigeria, focusing on WHO bacterial priority pathogens and ESKAPE organisms to assess community-level circulation of the most clinically significant antimicrobial-resistant bacteria. The specific objectives were to: (1) detect and characterize WHO priority pathogens and ESKAPE organisms in household wastewater samples across seven districts in Gombe; (2) determine AMR patterns and multidrug resistance (MDR) prevalence among isolated priority pathogens using standardized susceptibility testing; (3) investigate extended-spectrum beta-lactamase (ESBL) production and carbapenemase genes through phenotypic and molecular methods and (4) assess the geographic distribution of resistance patterns for priority pathogen circulation in household wastewater systems.

## Methods

### Study design and setting

This cross-sectional study was conducted between December 2024 and February 2025 in Gombe State, north-eastern Nigeria. The study focused on detecting and characterizing WHO bacterial priority pathogens and ESKAPE organisms in household wastewater within the 11 wards of Gombe Local Government Area (LGA), which comprises 46,112 households distributed across various districts [17]. Gombe LGA was selected due to its representative demographic profile, diverse population, mixing urban and rural communities, and its potential for environmental circulation of critical resistant organisms that pose threats to community health.

A multi-stage random sampling technique was employed to select specific communities and households, ensuring representation of different socio-economic and geographic characteristics. Seven districts were randomly selected from the state’s administrative divisions, representing both urban and rural settings. Within each district, proportional sampling was used to ensure representation proportional to population size. Two wards were randomly selected from each of the seven districts by balloting, and simple random sampling was applied to select households within each ward, minimizing selection bias and ensuring geographical representativeness.

### Sample size determination

Sample size was determined using Cochran’s formula for prevalence studies to ensure statistical validity [18,19]:

*n* = Z²*P*(1 − *P*)/e²

Where:

- Z=1.96 (95% confidence level)
- *P*=0.75 (estimated prevalence of MDR bacteria in wastewater based on [20])
- 1 − *P*=0.25 (proportion without the phenomenon)
- e=0.05 (5% margin of error).

This yielded an initial sample size of 288 households. Adjusting for a 10% non-response rate:

Final sample size = 288/(1 − 0.10) = 320 households.

### Sample collection

A total of 320 household wastewater samples were collected using a multi-stage sampling strategy across seven districts over the 3-month study period. Sampling locations were selected to represent different residential areas, socioeconomic strata and wastewater management systems. Sample collection was standardized to minimize temporal variation, with collections conducted during consistent early morning hours (6:00–9:00 AM) to capture similar household activities.

Twenty millilitres of wastewater samples were collected from each household into wide-mouthed, sterile plastic containers with screw-cap tops. Sample collection followed strict quality control measures, including GPS (Global Positioning System) coordinate recording, photographic documentation and standardized collection procedures. Containers were labelled with date, time and collection sites, then transported to the laboratory within 4 h using appropriate cold chain procedures, with chain-of-custody documentation ensuring sample integrity.

### Bacterial priority pathogen isolation and identification

Bacterial isolation was performed using standard conventional microbiological methods as described by Cheesbrough [21]. WHO critical priority pathogens and ESKAPE organisms were specifically targeted using selective isolation strategies. Water samples were first incubated in sterile peptone water overnight for enrichment. The enriched samples were then streaked onto differential selective media: Eosin Methylene Blue agar and MacConkey agar for Enterobacteriaceae, and cetrimide agar, with enhanced selectivity for *P. aeruginosa* recovery from environmental samples.

After 24 h of incubation at 37 °C, colonies were sub-cultured onto freshly prepared nutrient agar and incubated for 24 h to obtain pure isolates. This process was repeated until satisfactory pure isolates were obtained.

Morphological characteristics were observed after 24 h of growth, including cell shape, elevation, edge, consistency, colony surface and pigmentation. Gram staining with 100× optical microscopy was used to determine cellular morphology and classification.

Comprehensive biochemical testing was performed, including motility, indole, urease, citrate utilization, oxidase, methyl red, Voges–Proskauer and lactose fermentation tests. Results were compared with Bergey’s Manual of Determinative Bacteriology for accurate species identification [22]. Quality control included reference strains: *E. coli* ATCC 25922, *K. pneumoniae* ATCC 700603 and *P. aeruginosa* ATCC 27853.

We acknowledge that conventional biochemical identification has limitations compared with molecular methods (16S rRNA sequencing) or MALDI-TOF MS, particularly for environmental isolates. However, in our resource-limited setting, we implemented rigorous quality control measures, including American Type Culture Collection (ATCC) reference strains and comprehensive biochemical testing panels. Studies have demonstrated 85–95% concordance between biochemical and molecular methods for common Gram-negative pathogens when standardized protocols are followed [23,24]. While some degree of misidentification is possible, our identification of major species (*E. coli*, *Klebsiella* spp., *P. aeruginosa*) is considered reliable based on distinctive phenotypic characteristics and indirect confirmation through species-specific resistance gene detection.

### Antimicrobial susceptibility testing

Antimicrobial susceptibility testing (AST) was performed on Mueller–Hinton agar plates using the disc diffusion method (Kirby–Bauer technique) according to Clinical and Laboratory Standards Institute (CLSI) 2024 guidelines [25]. A standardized panel of 12 antimicrobial agents representing major antibiotic classes was selected: cefotaxime (CTX, 25 µg), cefuroxime (CXM, 30 µg), gentamicin (GEN, 10 µg), ceftriaxone (CRO, 30 µg), imipenem (IMP, 10 µg), ampiclox (ACX, 10 µg), ofloxacin (OFX, 5 µg), amoxicillin–clavulanate (AUG, 30 µg), cefepime (ZEM, 5 µg), nitrofurantoin (NF, 300 µg), nalidixic acid (NA, 30 µg) and levofloxacin (LEV, 5 µg). All antibiotic discs were procured from commercial suppliers providing CLSI-standardized potencies.

These antibiotics were selected to represent major classes (beta-lactams, fluoroquinolones, aminoglycosides and nitrofurans) relevant for treating Gram-negative infections in the study region, following CLSI 2024 guidelines for Gram-negative organism testing [25]. The panel was designed to assess community-level resistance trends in environmental surveillance rather than provide isolate-specific treatment guidance, though we acknowledge that not all antibiotics have equal clinical utility across all species tested.

Bacterial suspensions equivalent to 0.5 McFarland standard were prepared and evenly distributed on Mueller–Hinton agar plates. Antibiotic discs were aseptically placed using sterile forceps and incubated at 37 °C for 18 h. Inhibition zone diameters were measured to the nearest millimetre using a metre rule and interpreted according to CLSI breakpoints [25]. Isolates were classified as sensitive, intermediate or resistant.

### MDR definition and analysis

MDR was defined according to international consensus criteria as resistance to at least one agent in three or more antimicrobial categories [26,27]. The multiple antibiotic resistance (MAR) index was calculated using the formula: MAR index = Number of antibiotics to which isolate is resistant/Total number of antibiotics tested [28]. MAR index values >0.2 indicate high-risk contamination sources and significant antimicrobial pressure.

### ESBL detection

ESBL production was investigated using the double-disc synergy test (DDST) following European Committee on Antimicrobial Susceptibility Testing (EUCAST) guidelines [29]. CTX (30 µg), CRO (30 µg) and ceftazidime (30 µg) discs were placed 20 mm centre-to-centre from an AUG (30 µg) disc on Mueller–Hinton agar plates inoculated with test organisms.

ESBL production was confirmed by observing zone expansion or ‘keyhole’ effect between cephalosporin and clavulanate discs after 18 h incubation at 37 °C. Quality control used *K. pneumoniae* ATCC 700603 (ESBL-positive) and *E. coli* ATCC 25922 (ESBL-negative) reference strains.

### Molecular analysis

PCR was employed to detect key AMR genes in selected isolates representing different species and resistance phenotypes. DNA extraction was performed using commercial extraction kits following standard protocols, followed by PCR amplification targeting clinically significant resistance genes [30].

The molecular analysis targeted ESBL genes [beta-lactamase TEM gene (*blaTEM*), beta-lactamase SHV gene (*blaSHV*) and beta-lactamase CTX-M gene (*blaCTX-M*)] and carbapenemase genes [*K. pneumoniae* carbapenemase gene (*blaKPC*), Verona integron-encoded metallo-beta-lactamase gene (*blaVIM*) and New Delhi metallo-beta-lactamase gene (*blaNDM*)]. PCR reactions were performed in 25 µl volumes with initial denaturation at 95 °C for 5 min, followed by 35 cycles of denaturation (95 °C, 30 s), annealing (55–60 °C, 30 s) and extension (72 °C, 1 min), with final extension at 72 °C for 7 min. PCR products were analysed by gel electrophoresis on 1.5% agarose gels and visualized under UV light. Negative controls were included in each PCR run.

Due to resource constraints, molecular screening was performed on a limited subset of isolates: ten ESBL-producing Enterobacteriaceae (representing different species and geographic locations) and five carbapenem-resistant *P. aeruginosa* isolates. This represents a preliminary molecular characterization rather than comprehensive genetic surveillance of all resistant isolates.

### Statistical analysis

Statistical analysis was performed using R Statistics version 4.4.0. Descriptive statistics were calculated for all variables, including frequencies, percentages, medians and interquartile ranges. Chi-square tests were used for categorical variables, while Mann–Whitney U test or Kruskal–Wallis test was employed for continuous variables depending on data distribution.

Correlation analyses were performed to examine relationships between variables, such as the number of resistant antibiotics and MAR index values. Statistical significance was set at *P*<0.05, with confidence intervals calculated where appropriate.

### Ethical considerations

Ethical approval was obtained from the Gombe State Environmental Protection Agency (approval number: ES/GOSEPA/ADM/S/38 /V.I). The study protocol adhered to international guidelines for environmental research, ensuring all sampling activities complied with local regulations and community consent procedures. Environmental samples were collected from publicly accessible areas and with appropriate household permissions, with no personal identifiers recorded, and all data handled confidentially.

## Results

### Data summary

A total of 320 household wastewater samples were collected across seven districts in Gombe, Nigeria, yielding 402 Gram-negative bacterial isolates. Among these, the most frequently isolated WHO priority pathogens were *E. coli* (32.7%), *K. pneumoniae* (19.2%) and *P. aeruginosa* (11.2%). MDR, defined as resistance to ≥3 antibiotic classes, was observed in 80.8% (325/402) of isolates. Resistance was most prevalent in *Serratia marcescens* (100%), *Proteus mirabilis* (95.2%) and *Klebsiella oxytoca* (88.5%).

Resistance to key antibiotics included NF (58.7%), AUG (57.1%) and CTX (52.4%), with carbapenem resistance (IMP) detected in 25.4% of isolates. ESBL production was confirmed in 54.0% (127/235) of Enterobacteriaceae isolates, with the highest rates in *K. oxytoca* (73.1%), *K. pneumoniae* (51.9%) and *E. coli* (51.5%).

Molecular analysis of selected isolates revealed that blaCTX-M was present in 100% of ESBL-producing strains tested, while blaTEM and blaSHV were detected in 90% and 60%, respectively. Among carbapenem-resistant *P. aeruginosa*, blaVIM was detected in 60% and blaKPC in 20%, with no detection of blaNDM. High MDR prevalence was consistently observed across all seven districts, indicating widespread environmental dissemination of resistant pathogens.

### Bacterial species distribution and priority pathogen identification

A total of 402 Gram-negative bacterial isolates were recovered from household wastewater samples. *E. coli* was the most prevalent species (131 isolates, 32.7%), followed by *K. pneumoniae* (77 isolates, 19.2%) and *P. aeruginosa* (45 isolates, 11.2%). Additional priority pathogens detected included *Salmonella* spp*.* (44 isolates, 11%), *K. oxytoca* (26 isolates, 6.5%), *Enterobacter* species (31 isolates, 7.7%), *P. mirabilis* (21 isolates, 5.2%) and *S. marcescens* (19 isolates, 4.7%). The predominance of WHO bacterial priority pathogens and ESKAPE organisms among the most common isolates indicates widespread circulation of clinically significant antibiotic-resistant bacteria in the community.

### MDR patterns

MDR was alarmingly high across all bacterial species, with 325 of 402 isolates (80.8%) demonstrating resistance to three or more antibiotic classes. The highest MDR rates were observed in *S. marcescens* (100%), *P. mirabilis* (95.2%) and *K. oxytoca* (88.5%). Among other critical priority pathogens, *E. coli* showed 84.7% MDR prevalence, *K. pneumoniae* 79.2% and *P. aeruginosa* 73.3%. Despite the variation in MDR proportions, the chi-square test revealed no statistically significant difference across species (*P*>0.05).

### Antibiotic resistance profiles

Resistance to first-line antibiotics was widespread. The highest resistance rates were observed for NF (58.7%), AUG (57.1%) and CTX (52.4%). Concerning levels of resistance were also found for critical antibiotics, including ceftazidime (46%), CRO (42.9%) and IMP (41.3%). Carbapenem resistance was particularly troubling, as these are last-resort antibiotics, and was detected in 41.3% of isolates for imipenem. MDR isolates were resistant to an average of 7.8 antibiotics compared with only 1.4 antibiotics for non-MDR isolates (*P*<0.01).

Fig. 5 shows resistance percentages across 12 antibiotics for bacterial species isolated from household wastewater samples. *P. mirabilis* demonstrated universal resistance (100%) to most tested antibiotics except GEN, CXM and LEV. High resistance rates were observed for NF across multiple species, with *Salmonella* spp*.* and *Proteus* spp. showing 80–100% resistance. *E. coli*, the most prevalent isolate, exhibited notable resistance to AUG (75%), NF (65%) and CXM (65%). Priority pathogen *K. pneumoniae* showed concerning resistance patterns, with rates exceeding 50% for several first-line antibiotics. LEV demonstrated the lowest overall resistance rates across species, while NF and AUG showed the highest resistance frequencies.

### ESBL production

ESBL testing was performed on 235 Enterobacteriaceae isolates, revealing high rates of ESBL production across species. *E. coli* showed the highest ESBL prevalence, with 68 of 132 tested isolates (51.5%) being positive, followed by *K. pneumoniae* (40 of 77 isolates, 51.9%) and *K. oxytoca* (19 of 26 isolates, 73.1%). Overall, 127 of 235 tested isolates (54.0%) were ESBL-positive, indicating widespread dissemination of these resistance mechanisms in household wastewater.

Suspected carbapenemase-resistant *P. aeruginosa* isolates were selected based on preliminary identification using standard biochemical tests and confirmed by AST, which revealed resistance to carbapenems, such as imipenem.

### Molecular resistance gene analysis

PCR analysis (Table 1) of selected isolates revealed the genetic basis of observed resistance patterns. Among ESBL genes, *blaCTX-M* was universally present in all ten tested ESBL-producing isolates (100%), confirming its dominance in the study area. The *blaTEM* gene was detected in nine of ten isolates (90%), while *blaSHV* was found in six of ten isolates (60%).

**Table 1.** AMR genes detected by PCR in selected isolates from household wastewater, Gombe, Nigeria

Resistance gene	No. tested	Present, *n* (%)	Absent, *n* (%)
*bla_CTX-M*	10	10 (100%)	0 (0%)
*bla_SHV*	10	6 (60%)	4 (40%)
*bla_TEM*	10	9 (90%)	1 (10%)
*bla_NDM*	5	0 (0%)	5 (100%)
*bla_KPC*	5	1 (20%)	4 (80%)
*bla_VIM*	5	3 (60%)	2 (40%)

Carbapenemase gene analysis of five carbapenem-resistant *P. aeruginosa* isolates showed *blaVIM* in three isolates (60%) and *blaKPC* in one isolate (20%). *blaNDM* was not detected in the five tested isolates; however, this small sample size precludes conclusions about the prevalence of this gene in the broader isolate collection.

PCR screening of AMR genes in selected isolates: ESBL genes (*blaCTX-M*, *blaSHV* and *blaTEM*) were tested in ten ESBL-producing Enterobacteriaceae (*E. coli* and *K. pneumoniae*). Carbapenemase genes (*blaNDM*, *blaKPC* and *blaVIM*) were tested in five carbapenem-resistant *P. aeruginosa* isolates. Values represent the number and percentage of positive isolates out of the total tested.

### Geographic distribution

Analysis of MDR patterns across the seven districts in Gombe revealed a relatively uniform distribution of resistant bacteria, with all districts showing high MDR prevalence across bacterial species. *E. coli* demonstrated consistently high MDR rates, ranging from 70.6% in Bolari East to 100% in Dawaki, with all districts exceeding 70%. *K. pneumoniae* showed variable MDR rates across districts, from 36.4% in Jeka da Fari to 100% in Herwagana, while *P. aeruginosa* exhibited MDR rates between 50% and 100% across different locations.

Several species showed universal or near-universal MDR in multiple districts, including *S. marcescens* (100% in Pantami), *P. mirabilis* (100% in six districts) and *Salmonella* species (100% in three districts). The widespread distribution of high MDR rates across all geographic areas indicates systematic environmental contamination throughout the Gombe area rather than localized hotspots of resistance.

## Discussion

**Fig. 1.**
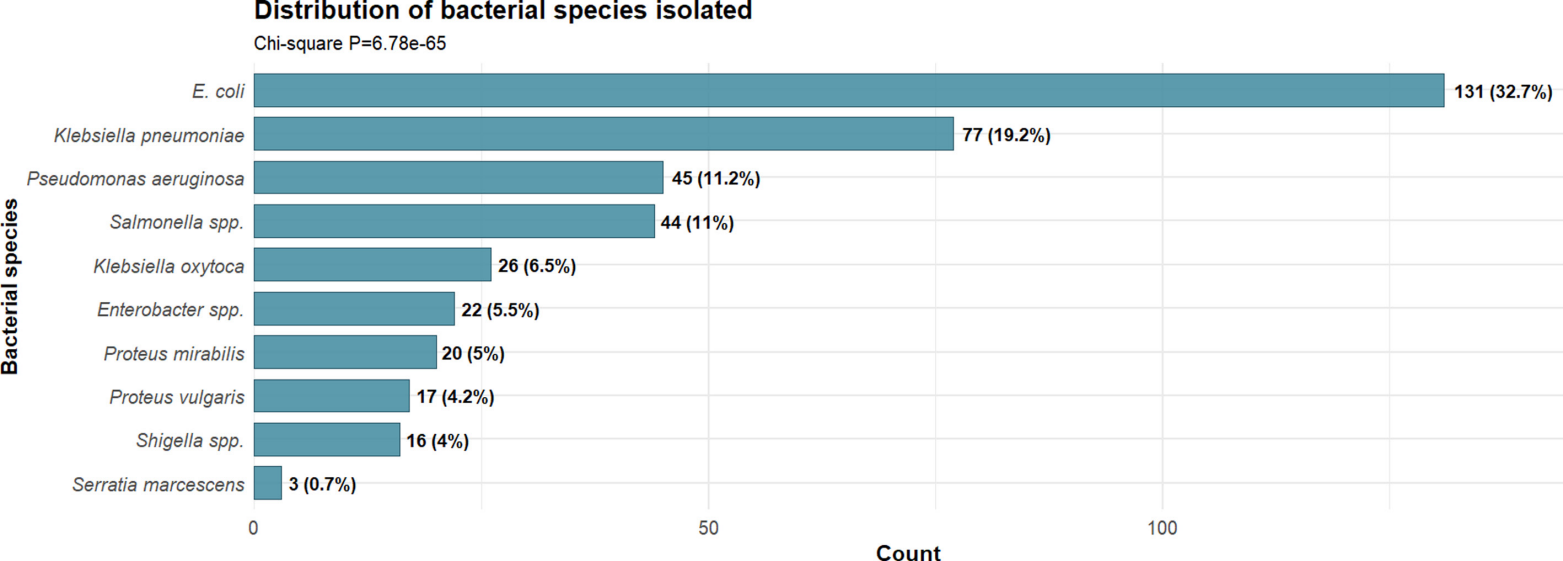
Species distribution of WHO priority pathogens isolated from household wastewater samples in Gombe. A total of 402 Gram-negative bacterial isolates were recovered from 320 household wastewater samples collected across seven districts in Gombe, Nigeria.

**Fig. 2.**
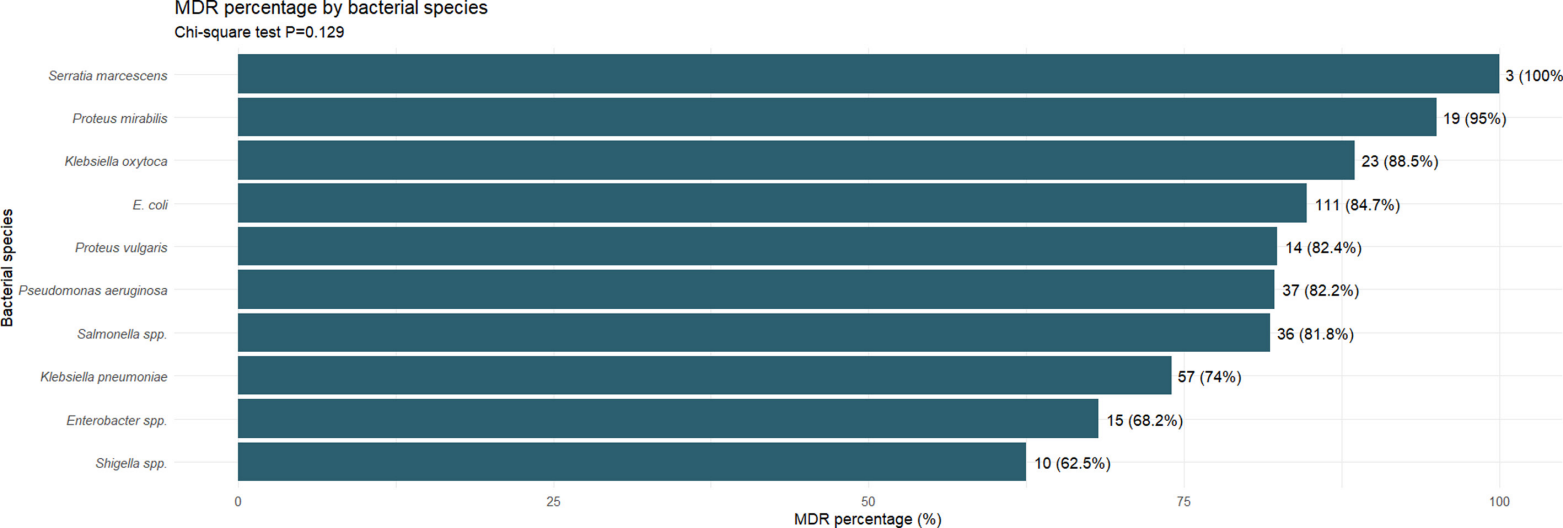
MDR prevalence among Gram-negative bacteria isolated from household wastewater in Gombe, Nigeria. MDR was defined as resistance to at least one agent in three or more antimicrobial categories. Sample sizes for each species were as follows: *E. coli* (*n*=131), *K. pneumoniae* (*n*=77), *P. aeruginosa* (*n*=45), *Salmonella* spp*.* (*n*=44), *Enterobacter* spp. (*n*=22), *K. oxytoca* (*n*=26), *P. mirabilis* (*n*=20), *S. marcescens* (*n*=3) and *Proteus vulgaris* (*n*=17).

**Fig. 3.**
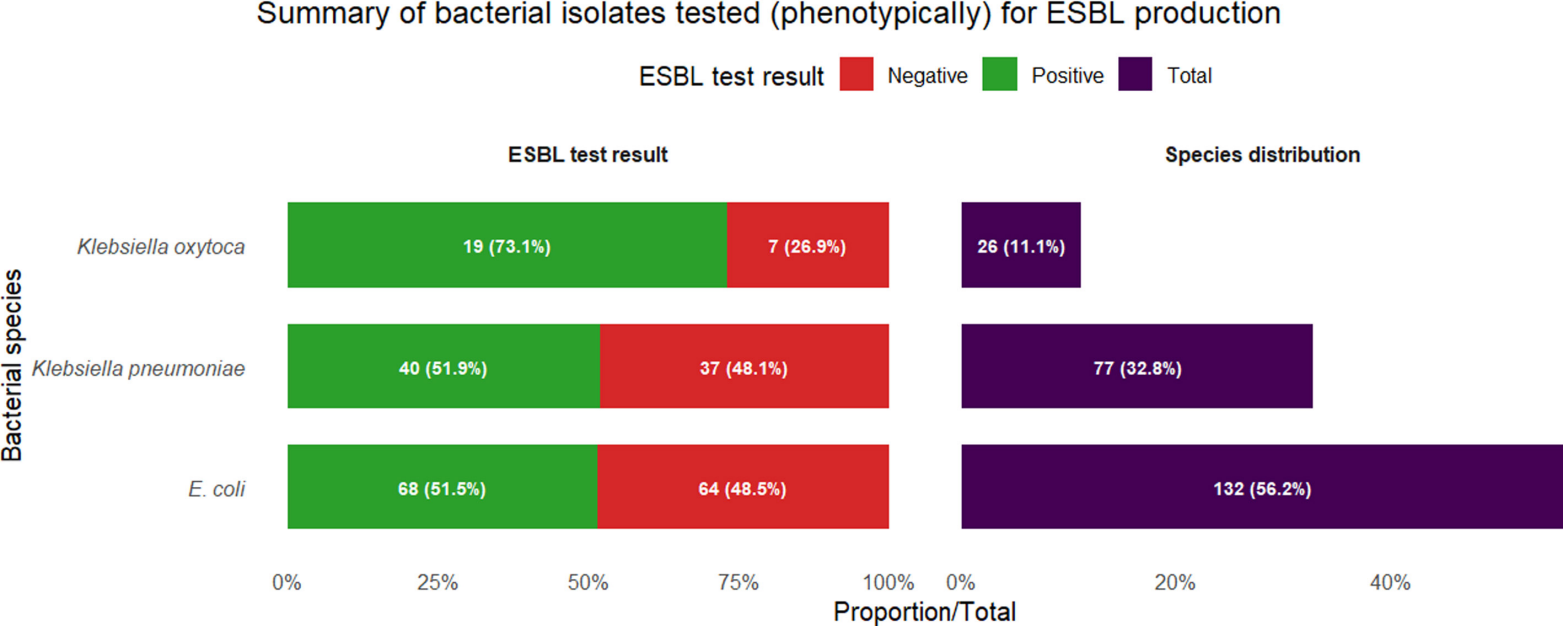
ESBL production among priority Enterobacteriaceae isolated from household wastewater in Gombe, Nigeria. ESBL testing was performed on 235 Enterobacteriaceae isolates using the DDST. Results by species were as follows: *E. coli*, 68/132 positive (51.5%); *K. pneumoniae*, 40/77 positive (51.9%); *K. oxytoca*, 19/26 positive (73.1%) and *Enterobacter* spp., 0/31 positive (0%). Overall, 127 of 235 tested isolates (54.0%) were ESBL-positive.

Our findings indicate that WHO bacterial priority pathogens and ESKAPE organisms are prevalent in household wastewater in Gombe. The dominance of *E. coli*, *K. pneumoniae* and *P. aeruginosa* as the most common isolates (Fig. 1), combined with high MDR prevalence (Fig. 2) and substantial ESBL production (Fig. 3), suggests considerable environmental circulation of resistant bacteria in the study area. However, it is important to recognize that environmental samples typically show different bacterial distributions and higher resistance rates than clinical samples, and further research is needed to establish the relationship between environmental detection and clinical infection risk in this setting.

**Fig. 4.**
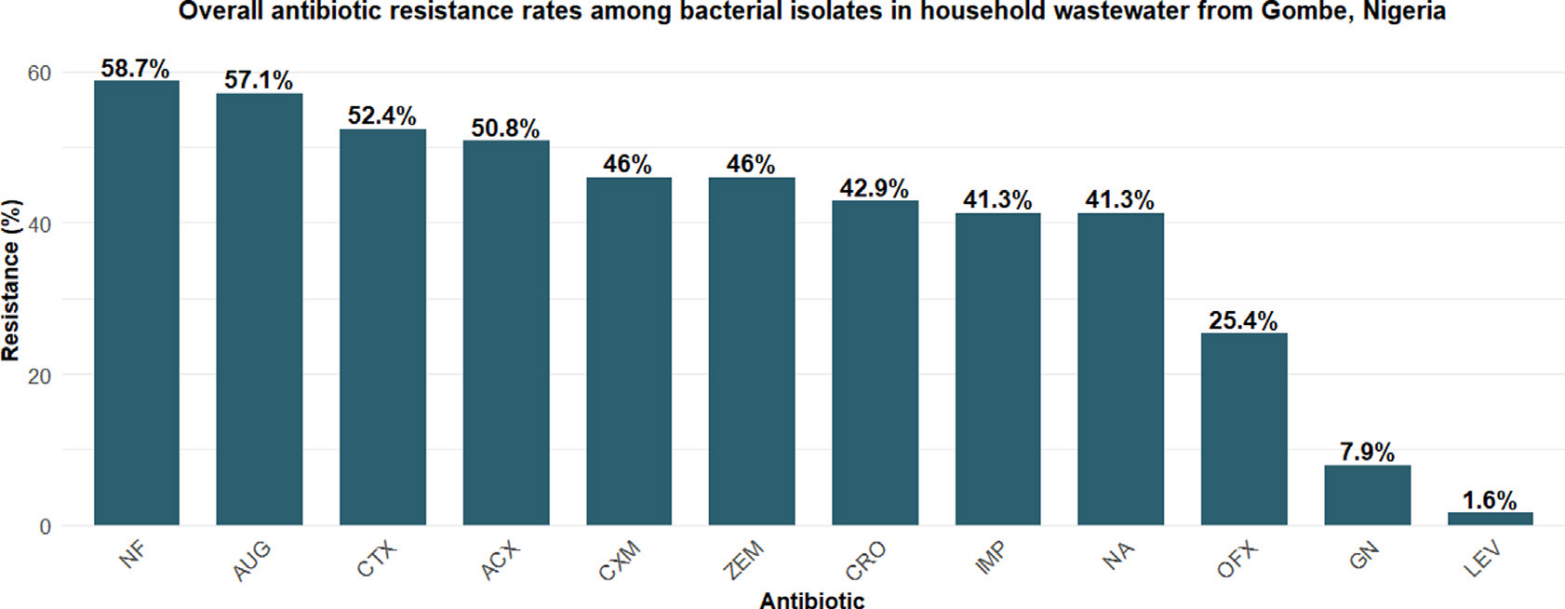
Overall antibiotic resistance rates among bacterial isolates from household wastewater in Gombe, Nigeria. Based on 402 isolates tested against 12 antibiotics using the disc diffusion method. Percentages represent the proportion of isolates resistant to each antibiotic.

**Fig. 5.**
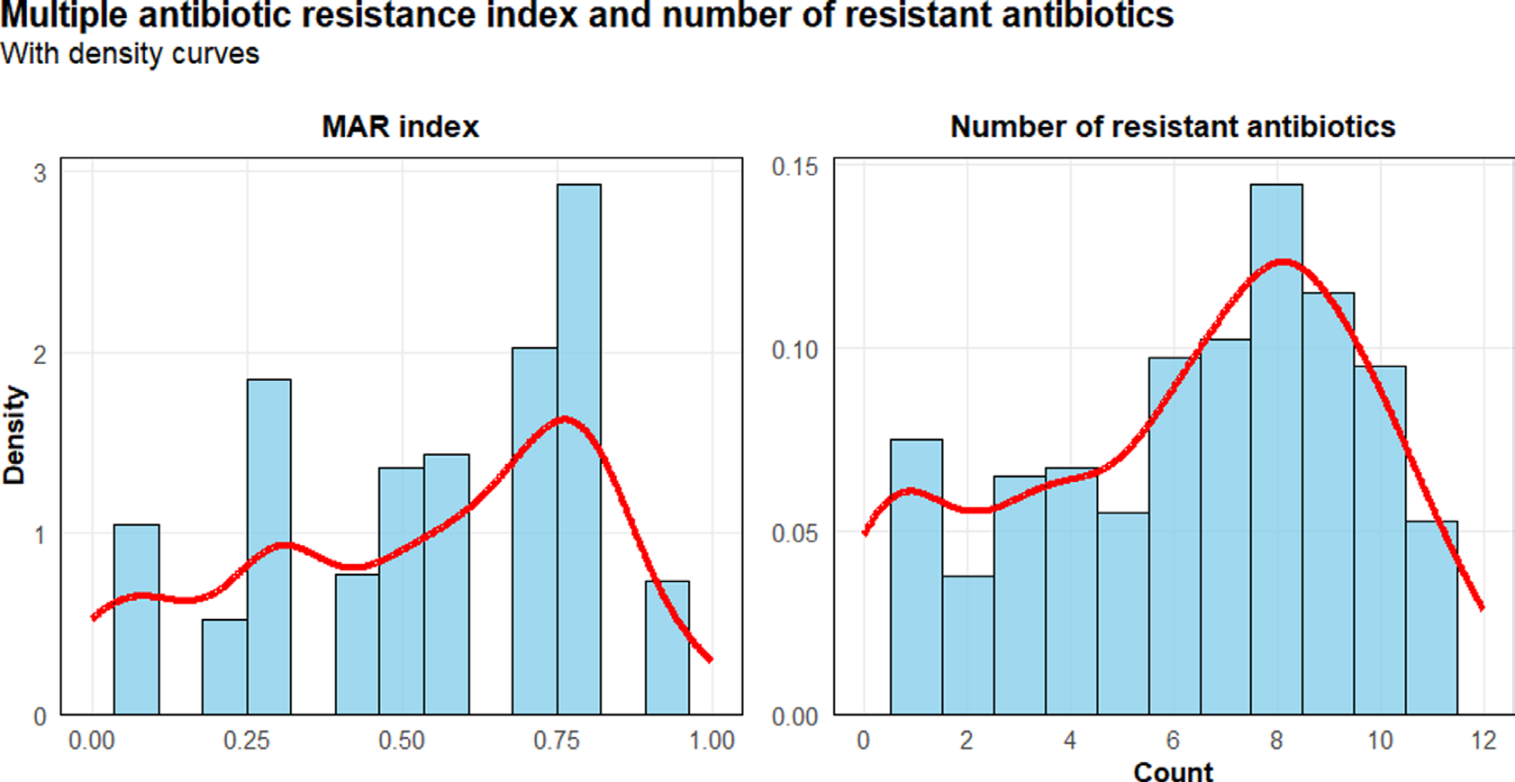
Distribution of MAR index values and number of resistant antibiotics among bacterial isolates from household wastewater in Gombe, Nigeria. Based on 402 isolates tested against 12 antibiotics. MAR index = number of antibiotics to which isolate was resistant/total number of antibiotics tested. MAR index values >0.2 indicate high-risk contamination sources. The histogram shows the frequency distribution of MAR index values (left panel) and number of resistant antibiotics per isolate (right panel).

The antibiotic resistance profiles demonstrate widespread resistance to first-line antibiotics, with the highest resistance rates observed for NF, AUGd and CTX (Fig. 4). Carbapenem resistance was detected in a substantial proportion of isolates, which is particularly concerning as carbapenems represent last-resort antibiotics for treating serious Gram-negative infections. The distribution of MAR index values (Fig. 5) further emphasizes the extent of multi-class resistance, with MDR isolates showing significantly higher numbers of resistances compared with non-MDR isolates.

**Fig. 6.**
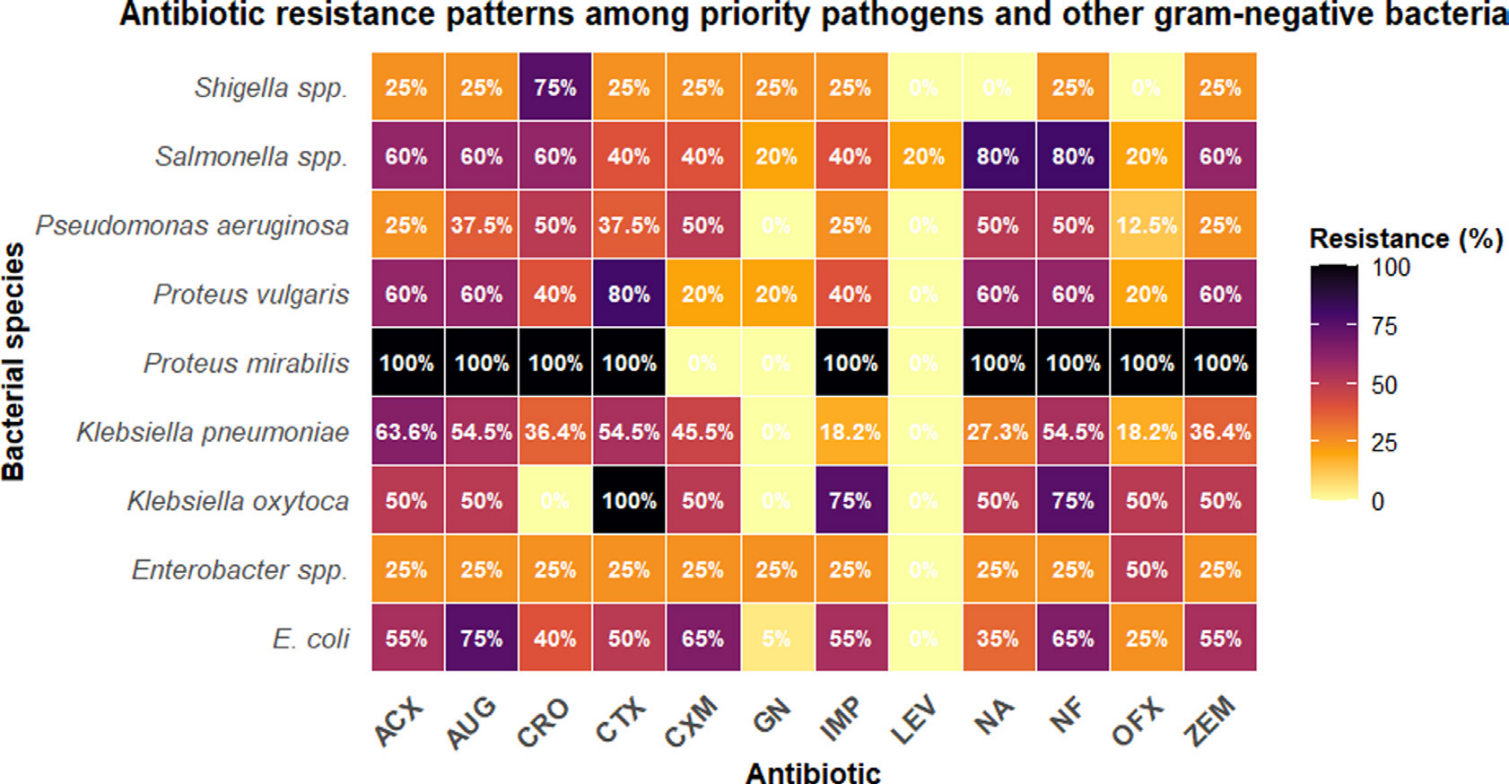
Antibiotic resistance patterns among priority pathogens and other Gram-negative bacteria isolated from household wastewater in Gombe, Nigeria. The heatmap shows resistance percentages for each bacterial species (rows) against 12 antibiotics (columns). Colour intensity indicates the percentage of resistant isolates, with darker red indicating higher resistance rates.

Species-specific resistance patterns (Fig. 6) reveal concerning profiles across multiple priority pathogens. *P. mirabilis* demonstrated near-universal resistance to most tested antibiotics, with notable exceptions for GEN, CXM and LEV. *E. coli*, the most prevalent isolate, exhibited high resistance rates to AUG, NF and CXM. Priority pathogen *K. pneumoniae* showed resistance rates exceeding half of tested isolates for several first-line antibiotics, limiting therapeutic options for infections caused by this organism.

ESBL production was confirmed in over half of tested Enterobacteriaceae, with particularly high rates in *K. oxytoca*, *K. pneumoniae* and *E. coli* (Fig. 3). Molecular confirmation (Table 1) revealed universal presence of *blaCTX-M* genes in the tested ESBL-producing isolates, along with high detection rates for *blaTEM* and moderate detection of *blaSHV*. The dominance of *blaCTX-M* aligns with global trends, where CTX-M enzymes have emerged as the most prevalent ESBL type worldwide [31,32]. Among carbapenem-resistant *P. aeruginosa* isolates tested, *blaVIM* was the most frequently detected carbapenemase gene, followed by *blaKPC*, while *blaNDM* was not detected in the limited number of isolates screened.

However, given our small molecular screening sample, these gene prevalence figures should be interpreted cautiously. The absence of *blaNDM* in our small sample cannot be considered evidence of its absence in the broader community, particularly given reports of NDM-type carbapenemases in southern Nigeria and East Africa [33,34]. Comprehensive molecular surveillance with larger sample sizes would be required to accurately characterize resistance gene epidemiology in this setting.

The 80.8% overall MDR prevalence observed in this study is high; however, direct comparisons with other studies should be made cautiously due to methodological differences in sample collection, bacterial identification methods, AST approaches and MDR definitions. Environmental surveillance studies often report higher resistance rates than clinical studies due to differences in sampling matrices and selection pressures.

Our ESBL prevalence falls within the reported range for West and Central Africa, though at the upper end [35]. A systematic review and meta-analysis found substantially lower global prevalence of ESBL-producing Enterobacteriaceae in wastewater [36]. While our prevalence exceeds this global estimate, the difference may partially reflect methodological variations, including our use of conventional biochemical identification, differences in ESBL detection methods and geographic/temporal factors. For comparison, a study in Burkina Faso found even higher ESBL rates in healthcare wastewater samples [37], though this represents a fundamentally different sample type with different selective pressures.

Our findings align with previous environmental surveillance reports from Nigeria and other sub-Saharan African countries, documenting high prevalence of ESBL-producing Enterobacteriaceae and multidrug-resistant *P. aeruginosa* in wastewater and surface water samples [14,20, 37, 38]. The overall MDR prevalence observed in our study falls within the upper range reported for the region, reflecting a substantial environmental AMR burden. These comparisons suggest geographical variability in resistance mechanisms and emphasize the need for harmonized environmental surveillance approaches with standardized methodologies to enable meaningful regional comparisons.

Globally, environmental AMR monitoring highlights widespread dissemination of beta-lactamase genes, although regional variation is notable due to differences in antibiotic consumption, sanitation infrastructure and local surveillance practices [39,41]. International wastewater surveillance programmes have increasingly recognized the value of environmental monitoring for AMR, with recent systematic reviews emphasizing wastewater as an early-warning system for emerging resistance [42,43].

**Fig. 7.**
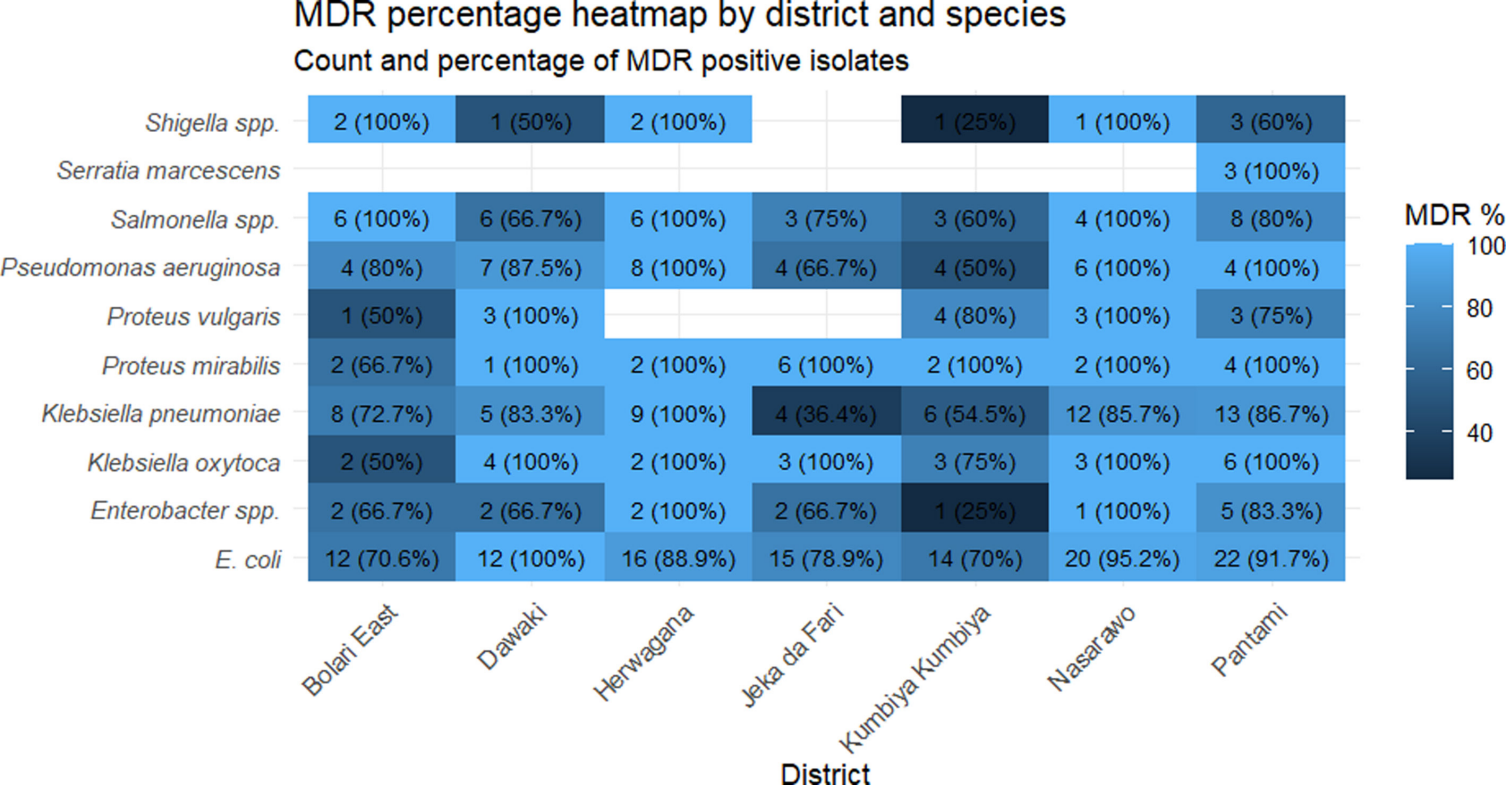
Geographic distribution of MDR among priority pathogens isolated from household wastewater across seven districts in Gombe, Nigeria. The figure shows MDR prevalence (percentage of isolates resistant to ≥3 antibiotic classes) for each bacterial species across the seven districts. Sample sizes varied by district and species, with total isolates per district ranging from 43 to 71. *E. coli* was isolated from all seven districts (*n*=131 total), *K. pneumoniae* from all districts (*n*=77 total) and *P. aeruginosa* from six districts (*n*=45 total). Bars represent the percentage of MDR isolates for each species within each district.

The geographic distribution of MDR patterns (Fig. 7) across the seven districts in Gombe revealed a relatively uniform distribution of resistant bacteria, with all districts showing high MDR prevalence. This widespread pattern indicates systematic environmental contamination throughout the study area rather than localized hotspots. Similar systematic contamination patterns have been observed in other urban areas with limited wastewater treatment infrastructure [44,45], suggesting that interventions must be comprehensive rather than targeted to specific high-risk areas.

Our study design cannot identify specific sources or pathways of environmental contamination. Potential contributing factors may include limited wastewater treatment infrastructure, antibiotic-use patterns in the community, possible connections between healthcare and community wastewater systems, and point sources such as pharmaceutical facilities. Studies from similar resource-limited settings have shown that pharmaceutical wastewater can be a significant source of resistant bacteria, with one Nigerian study reporting very high MDR prevalence in pharmaceutical wastewaters [38]. Limited wastewater treatment infrastructure has been documented in many low- and middle-income countries [46,47], which may contribute to environmental dissemination of resistant bacteria. Longitudinal studies with source tracking would be needed to establish the relative contributions of different sources and inform targeted interventions.

The high ESBL prevalence in household wastewater suggests that community-acquired infections in Gombe may increasingly involve organisms with limited oral treatment options. This environmental prevalence substantially exceeds the global community ESBL carriage rate reported in healthy individuals [48], though direct comparisons between environmental detection and human colonization should be made cautiously. Systematic reviews of ESBL prevalence in sub-Saharan Africa and Nigeria have documented high rates across hospital and community settings [49,50], and our environmental data contribute additional evidence of widespread ESBL dissemination in the region.

For common infections, such as urinary tract infections caused by *E. coli*, high ESBL prevalence means that standard oral antibiotics may have reduced effectiveness, potentially necessitating more expensive injectable treatments or hospitalization. However, our environmental surveillance data cannot directly predict clinical treatment outcomes, and integrated clinical–environmental surveillance would be needed to establish these relationships.

The detection of carbapenem-resistant bacteria in household wastewater suggests environmental circulation of organisms carrying last-resort antibiotic resistance. However, our cross-sectional study design cannot determine the sources, temporal trends or transmission dynamics of these organisms. Further research is needed to establish whether detected organisms represent spillovers from healthcare settings, community acquisition, environmental selection or other sources, and to assess the relationship between environmental detection and clinical infection risks.

This study provides preliminary environmental surveillance data from a resource-limited setting using conventional microbiological methods. While molecular identification and MIC-based susceptibility testing represent gold standards, our approach demonstrates the feasibility of wastewater-based AMR monitoring in settings where advanced laboratory infrastructure is limited. Our findings validate the approach of environmental monitoring for AMR while demonstrating that environmental resistance levels can exceed clinical estimates, particularly in settings with limited healthcare surveillance infrastructure.

These results underscore the need for: (1) establishing systematic environmental surveillance programmes with standardized methodologies; (2) strengthening laboratory capacity for molecular identification and resistance gene detection; (3) conducting larger scale molecular surveillance to accurately characterize resistance mechanisms; (4) improving wastewater treatment infrastructure; (5) implementing enhanced antimicrobial stewardship in both healthcare and community settings and (6) conducting longitudinal studies with source attribution to track resistance trends and transmission dynamics over time.

Our data contribute baseline information for Nigerian environmental AMR surveillance and highlight the importance of integrating environmental monitoring into broader One Health surveillance frameworks. However, findings should be interpreted as hypothesis-generating data, requiring validation with more rigorous molecular methods and broader geographic sampling, rather than as definitive prevalence estimates or evidence of specific transmission pathways.

## Study limitations and future directions

This study has some important limitations that constrain the strength of our conclusions and should guide interpretation of our findings. As a cross-sectional study, our research provides a snapshot of resistance prevalence at a single time point but cannot establish temporal trends, identify sources of contamination or demonstrate causality. This design limits our understanding of seasonal variations and resistance evolution over time. Claims about infection control failures, containment strategy effectiveness or directionality of resistance spread cannot be supported by our study design. Future longitudinal studies should track resistance patterns over time, identify peak transmission periods and incorporate source attribution studies with clinical correlation to establish transmission dynamics and evaluate intervention effectiveness.

The use of conventional biochemical identification rather than molecular methods (16S rRNA sequencing) or MALDI-TOF MS represents a limitation, as some environmental isolates may be misidentified. Future studies should incorporate molecular species confirmation to improve accuracy. However, our quality-controlled biochemical approach with ATCC reference strains provides reasonable estimates for the most common Gram-negative pathogens, particularly for environmental surveillance purposes, where population-level resistance trends are the primary focus rather than precise species-level prevalence.

AST was performed using disc diffusion rather than broth microdilution MIC testing. While disc diffusion is a CLSI-validated method widely used in clinical and environmental surveillance, MIC determination represents the gold standard for detecting carbapenem resistance and would provide more precise resistance characterization. Studies report variable categorical agreement (55–95%) between disc diffusion and broth microdilution for carbapenems depending on organism and carbapenemase type, with carbapenems being particularly challenging compared with other antibiotic classes [41,51, 52]. Our carbapenem resistance data should therefore be interpreted as surveillance indicators of concerning resistance trends rather than precise prevalence estimates. Future studies incorporating automated MIC testing or molecular detection of carbapenemase genes across all isolates would provide enhanced accuracy in resistance quantification.

The molecular analysis in this study was limited in both scope and sample size, representing a major limitation. Only ten of 127 ESBL-producing isolates and five carbapenem-resistant *P. aeruginosa* isolates underwent PCR screening, precluding robust epidemiological conclusions about resistance gene prevalence. Additionally, our gene panel was limited and did not include OXA-48-like carbapenemases (*blaOXA-48* [oxacillinase-48] and *blaOXA-181* [oxacillinase-181]), which are increasingly prevalent in Africa, or other clinically relevant resistance determinants, such as AmpC beta-lactamases (plasmid-mediated cephalosporinases). The absence of *blaNDM* in our small sample cannot be interpreted as evidence of its absence in the community. Future studies should employ comprehensive molecular surveillance with whole-genome sequencing across representative isolate collections to accurately characterize resistance mechanisms and track gene dissemination patterns. Expanded molecular epidemiology would also enable investigation of clonal relationships and horizontal gene transfer dynamics in environmental settings.

The lack of parallel clinical resistance data limits our ability to establish direct transmission links between environmental and clinical isolates. Environmental resistance rates do not directly translate to clinical infection risks, and our findings cannot determine whether environmental detection predicts clinical treatment failures. Future studies should employ whole-genome sequencing to trace resistance gene flow between environmental and clinical compartments and conduct integrated surveillance linking environmental detection with clinical outcomes.

This study represents preliminary environmental surveillance data from a resource-limited setting. The use of conventional biochemical identification and disc diffusion susceptibility testing, while appropriate for resource-limited settings, introduces uncertainty compared with molecular identification and MIC determination. Comparisons with clinical surveillance data and systematic reviews should be made cautiously, given the fundamental differences in sample types, methodologies and study objectives. Our findings should be interpreted as hypothesis-generating data that highlight the need for enhanced surveillance infrastructure and more comprehensive molecular epidemiological studies in the region, rather than definitive evidence of resistance prevalence or public health system performance.

## Conclusion

This cross-sectional study provides preliminary evidence of WHO priority pathogens and ESKAPE organisms in household wastewater from Gombe, Nigeria, with notable rates of MDR and ESBL production. While methodological limitations, such as conventional biochemical identification, disc diffusion susceptibility testing and limited molecular screening, constrain the strength of our conclusions, our findings indicate the environmental presence of resistant bacteria in the study area that warrants further investigation.

Environmental resistance levels do not directly translate to clinical infection risks, and our study design cannot establish sources, transmission dynamics or temporal trends. However, this work contributes baseline data for Nigerian environmental AMR surveillance in an underrepresented region and demonstrates both the feasibility and limitations of wastewater-based monitoring in resource-limited settings. These findings underscore the urgent need for enhanced surveillance infrastructure, comprehensive molecular characterization and integrated clinical–environmental research to better understand and address AMR in community settings. Future studies should employ more rigorous molecular methods, larger sample sizes and longitudinal designs to validate these preliminary observations and inform evidence-based public health interventions.

## Supplementary material

10.1099/acmi.0.001100.v3 Uncited Supplementary Material 1.

## Abbreviations

ACX: ampiclox
AMR: antimicrobial resistance
AST: antimicrobial susceptibility testing
ATCC: American Type Culture Collection
AUG: amoxicillin–clavulanate
blaCTX-M: beta-lactamase CTX-M gene
blaKPC: *Klebsiella pneumoniae* carbapenemase gene
blaNDM: New Delhi metallo-beta-lactamase gene
blaSHV: beta-lactamase SHV gene
blaTEM: beta-lactamase TEM gene
blaVIM: Verona integron-encoded metallo-beta-lactamase gene
CLSI: Clinical and Laboratory Standards Institute
CRO: ceftriaxone
CTX: cefotaxime
CXM: cefuroxime
DDST: double-disc synergy test
ESBL: extended-spectrum beta-lactamase
ESKAPE: *Enterococcus faecium*, *Staphylococcus aureus*, *Klebsiella pneumoniae*, *Acinetobacter baumannii*, *Pseudomonas aeruginosa* and *Enterobacter* spp.
EUCAST: European Committee on Antimicrobial Susceptibility Testing
GEN: gentamicin
GPS: Global Positioning System
IMP: imipenem
LEV: levofloxacin
LGA: Local Government Area
MAR: multiple antibiotic resistance
MDR: multidrug resistance
NA: nalidixic acid
NF: nitrofurantoin
OFX: ofloxacin
WHO: World Health Organization
ZEM: cefepime
